# Nutritional and Chemical Composition of *Sargassum zhangii* and the Physical and Chemical Characterization, Binding Bile Acid, and Cholesterol-Lowering Activity in HepG2 Cells of Its Fucoidans

**DOI:** 10.3390/foods11121771

**Published:** 2022-06-15

**Authors:** Peichun Lin, Suhua Chen, Siyan Zhong

**Affiliations:** 1 School of Chemistry and Environment, Guangdong Ocean University, Zhanjiang 524088, China; 2111911013@stu.gdou.edu.cn; 2 School of Food Science and Technology, Guangdong Ocean University, Zhanjiang 524088, China; zhongxiaojun21@stu.gdou.edu.cn

**Keywords:** *Sargassum zhangii*, nutritional and chemical composition, fucoidan, physicochemical characterization, bile acid, cholesterol-lowering activity

## Abstract

Fucoidan is a marine sulfated polysaccharide that is rich in *Sargassum* and has a wide range of biological activities. In this study, the chemical composition and bile acid binding ability of six crude fucoidans were compared, the nutrition and chemical composition of *Sargassum zhangii* were analyzed, and fucoidan from *Sargassum zhangii* was extracted and purified. The purified fractions (ZF1, ZF2, and ZF3) were analyzed by physicochemical characterization, and the ability of binding bile acid and cholesterol lowering in HepG2 cells were evaluated. The results showed that the contents of sulfate in crude fucoidan from *Sargassum Zhangii* (ZF) was as high as13.63%. Its ability of binding bile acid was better than other five crude fucoidans. *Sargassum zhangii* was a kind of brown seaweed with high carbohydrate, and low fat and rich in minerals. The sulfate content of ZF1, ZF2, and ZF3 was 3.29%, 19.39%, and 18.89% respectively, and the molecular weight (Mw) was 4.026 × 10^5^, 2.893 × 10^5^, and 3.368 × 10^5^, respectively. Three fucoidans all contained the characteristic absorption bands of polysaccharides and sulfate groups and were rich in fucose. Three fucoidans can bind to bile acid, and ZF2 showed the best binding capability. In vitro experiments showed that ZF1, ZF2, and ZF3 could reduce intracellular total cholesterol (TC) content in HepG2 cells without affecting their viability. ZF2 showed the best ability to reduce TC.

## 1. Introduction

Hyperlipidemia is a disease caused by excessive intake of cholesterol beyond the range of human metabolism and the accumulation of unmetabolized cholesterol in the blood vessels [1]. It can cause a series of cardiovascular and cerebrovascular diseases, such as coronary heart disease, stroke, and so on. Hyperlipidemia killed an estimated 3.9 million people worldwide in 2017, half of them from China and other Southeast Asian countries [2]. Hyperlipidemia is mainly divided into three categories, including hypercholesterolemia. In the past 40 years, the cholesterol level of Chinese residents may have been higher in the world, leaping ahead of many developed countries [2]. Hypercholesterolemia is mainly treated from three aspects: diet, lifestyle changes, and drugs. One of the drug treatments is to enhance reverse cholesterol transport and transport plasma cholesterol back to the liver for catabolism. The other is to enhance the conversion of cholesterol to bile acid in the liver [3,4]. Statins, cholic acid chelating agents, niacin, bates, and other drugs for the treatment of high cholesterol have side effects on human health. Therefore, it is particularly important to find a non-toxic and edible substitute with homologous medicine and food.

Genus *Sargassum* represents Phaeophyta and plays an important role as a food source and habitat in the tropical waters of marine ecosystems [5]. *Sargassum* spp. was recorded in *Shennong Materia Medica Classic* and *Compendium of Materia Medica*. They were used in Traditional Chinese Medicine to treat a variety of diseases [6]. *Sargassum* was found to be rich in new bioactive compounds. Among these bioactive compounds, fucoidan has received considerable attention because of its proven health benefits [5]. In recent years, polysaccharides from a variety of natural sources have been proven to show a wide range of biological activities with few side effects. These polysaccharides have gradually developed into functional foods with specific therapeutic effects [7]. For example, fucoidan could significantly reduce plasma total cholesterol (TC), triglyceride (TG), and low-density lipoprotein cholesterol (LDL-C); increase high-density lipoprotein cholesterol (HDL-C); and effectively reduce the level of serum lipid and effectively prevent lipid accumulation in the liver [1,8,9,10,11]. In addition, fucoidan has a wide range of biological properties, such as antioxidant [12], immune regulation [13], antiviral [14], anticoagulant [15], and so on. Fucoidan mainly contains L-fucose and sulfate as well as a small amount of other monosaccharides, such as xylose, galactose, mannose, and rhamnose [16]. Fucoidan is a kind of water-soluble heteropolysaccharide, which is abundant in *Sargassum* spp. [17]. *Sargassum* is mainly distributed in tropical and subtropical waters and usually grows on rocky reefs. For example, *Sargassum* is rich in species in the Zhanjiang sea area, with an annual output of about 100,000 piculs (dry products) [18].

In this study, six *Sargassum* spp. were collected from Zhanjiang, China. Six crude fucoidans were extracted by ultrasonic-assisted hot water extraction, and their bile acids binding ability was compared. The nutrition and chemistry of *Sargassum zhangii* were analyzed, and the crude fucoidan from *Sargassum zhangii* was classified and purified. The physicochemical characterization of purified fucoidan fractions, the ability to bind bile acids, and the cholesterol-lowering effect on HepG2 cells were studied. Our study provides a theoretical basis for the application of fucoidan in adjuvant cholesterol-lowering therapy.

## 2. Materials and Methods

### 2.1. Samples and Chemicals

The six Sargassum (*Sargassum henslowianum*, *Sargassum zhangii*, *Sargassum hemiphyllum*, *Sargassum naozhouense*, *Sargassum integerrimum*, and *Sargassum wightii*) came from the sea area in Zhanjiang City, Guangdong Province, China. These Sargassum were identified by Professor Xie Enyi of Guangdong Ocean University. The Sargassum were washed with clean water and dried naturally. Then, they were dried at 50 °C for 1 h, ground into powder, and passed through the 80-mesh sieve. Sargassum powder was packaged and stored in an ultra-low temperature refrigerator (Merit, Kaltis, Waltham, MA, USA) at −80 °C. Taurocholic acid, cholic acid, and glycocholic acid were purchased from Aladdin (Shanghai, China). DEAE cellulose was obtained from Guangzhou Dingguo biological Co., Ltd. (Guangzhou, China). Seven standard monosaccharides, including fucose, glucose, mannose, galactose, rhamnose, xylose, and arabinose, were purchased from Shanghai Yuanye Biotechnology Co., Ltd. (Shanghai, China). DMEM medium, fetal bovine serum (FBS), penicillin/streptomycin, and 0.25% trypsin–EDTA were obtained from Gibco (Grand Island, NY, USA). Sephacryl Smur300 HR gel, glucan, 3-(4,5-Dimethylthiazol-2-yl)-2,5-diphenyltetrazolium bromide (MTT), and dimethyl sulfoxide (DMSO) were purchased from Sigma (City of Saint Louis, LA, USA). All other reagents used in the experiment were of analytical grade and are commercially available.

### 2.2. Extraction of Crude Fucoidans from Six Sargassum

According to our previous method, crude fucoidans from *Sargassum* spp. were extracted [19]. According to the ratio of material to liquid at 1:30, dried sargassum was added to distilled water. The pH of the solution was adjusted to 6.0. The samples were treated with ultrasonic (KQ-500DB, Kunshan Ultrasonic instrument Co., Ltd., Kunshan, China) at 350 W for 50 min and reacted in a constant temperature water bath at 80 °C for 3.5 h. The large particle residue was removed with 200-mesh filter cloth and centrifuged at 4000 rpm/min for 10 min. The supernatant was added with anhydrous ethanol to a concentration of 30% and then centrifuged at 4000 rpm/min for 10 min. The supernatant was added with anhydrous ethanol until the concentration was 75%. After standing overnight at 4 °C, the solution was centrifuged at 4000 rpm/min for 10 min to obtain precipitation, which was washed twice with anhydrous ethanol, acetone, and ether, respectively. We added Sevage reagent and shook violently with the solution obtained in the previous step for 20 min, then centrifuged for 10 min at 4000 rpm/min, took the supernatant, and repeated this operation 6 times. The supernatant was dialyzed in distilled water for 48 h and concentrated in vacuum freeze drying to obtain crude fucoidan. The crude fucoidans from *Sargassum henslowianum*, *Sargassum zhangii*, *Sargassum hemiphyllum*, *Sargassum naozhouense*, *Sargassum integerrimum*, and *Sargassum wightii* were identified as HenF, ZF, HemF, NF, IF, and WF.

### 2.3. Analysis of Chemical Constituents of Fucoidans

The total sugar was determined by anthrone colorimetry [20]. In total, 0, 0.1, 0.2, 0.3, 0.4, and 0.5 mL of glucose solution with concentration of 100 μg/mL were absorbed into the test tube, and distilled water was added to make the total volume of 1 mL. Then, 4 mL anthrone reagent was added in ice water bath, then boiling water bath for 10 min, cooled to room temperature, placed at room temperature for 10 min, and the absorbance was measured at 620 nm. The standard curve was drawn with glucose concentration as abscissa and absorbance value as ordinate. Next, 1 mL 0.025 mg/mL fucoidan solution was added in the test tube, and the absorbance of fucoidan solution was measured, and the total sugar content was calculated according to the standard curve. The content of fucose was determined by cysteine hydrochloric acid method [21]. Accurately, we next took 0.2, 0.4, 0.6, 0.8, and 1.0 mL of 40 μg/mL L-fucose solution in a test tube, added distilled water to make up to 1 mL, and slowly added 4.5 mL of sulfuric acid dropwise, placed it in an ice-water bath for 1 min, and followed with a boiling water bath for 10 min. After cooling to room temperature, 0.1 mL of 3% L-cysteine hydrochloride was added dropwise to each tube, and the tubes were placed in a water bath at 37 °C for 90 min. After cooling to room temperature, the absorbance values A1 and A2 were measured at 396 nm and 427 nm, respectively. We took the L-fucose concentration as the abscissa and (A1–A2) as the ordinate to make a standard curve. Then, 1 mL 100 μg/mL fucoidan solution was added to the test tube, and after the above experimental steps, the absorbance was determined at 396 nm and 427 nm, respectively. The content of L-fucose was calculated according to the standard curve. The content of sulfate was determined by BaCl_2_ gel method [22]. We next added 0.04, 0.08, 0.12, 0.16, and 0.20 mL of 0.6 mg/mL sulfate solution to the test tube, respectively, and added 1 mol/L HCl to make up to 0.2 mL and 3% TCA 3.8 mL. This was repeated twice to obtain two groups of test tubes. We then added 1 mL of 1% barium chloride 0.5% gelatin solution to each tube of the first group, added 1 mL of 0.5% gelatin solution to each tube of the second group, and let stand for 15 min at room temperature. The first group of absorbance values measured at 360 nm was recorded as A3, and the second group was recorded as A4. Next, we took the sulfate concentration as the abscissa and the difference between the two groups of absorbance values (A3–A4) as the ordinate to draw a standard curve. Then, we dissolved 15 mg fucoidan with 1 mol/L HCl and fixed the volume to 10 mL, sealed the tube in boiling water bath for 5 h, cooled to room temperature, and added 0.2 mL to the test tube. According to the above method, the difference of absorbance value was substituted into the standard curve to calculate the sulfate concentration.

### 2.4. Bile Acid Binding Capacity Assay

According to the method of Long et al. [23], Deng et al. [24], and Feng et al. [25], we proceeded with a slight modification: 0.3 mmol/L taurocholic acid, cholic acid, and glycocholic acid solution WAS prepared with PBS as mother liquid. We then took 0, 0.1, 0.5, 1.0, 1.5, and 2.5 mL of the above solutions in a 10 mL plug test tube, added PBS to 2.5 mL, then added 7.5 mL 60% H_2_SO_4_, performed a water bath at 70 °C for 20 min, and cooled in an ice water bath for 5 min. The absorbance was measured at 387 nm. The standard curve was made according to the concentration of bile acid and absorbance. The fucoidan solution reacted with 1 mL 0.01 mol/L hydrochloric acid solution at 37 °C for 2 h. The pH was adjusted to 7.6 with 0.1 mol/L NaOH. Next, 4 mL bile acid solution was added to each sample and oscillated at 37 °C for 2 h. After 2 h, the supernatant was centrifuged at 4000 rpm/min for 20 min, and the concentration of bile acid in the supernatant was determined by colorimetry. The absorbance of 2.5 mL sample solution was determined according to the standard curve. Bile acid binding rate = (1 − *c*_1_/*c*_0_) × 100%; *c*_1_ represents the bile acid concentration of the fucoidan solution, mmol/L; and *c*_0_ indicates the bile acid concentration of the blank solution, mmol/L.

### 2.5. Analysis of Basic Components of Sargassum zhangii

The dry-ashing method at 550 °C was employed to measure ash content. The protein was detected by trace Kjeldahl nitrogen determination [26]. Soxhlet extraction was used to detect crude fat. Carbohydrates were detected by subtraction [27]. The neutral washing method was performed to detect dietary fiber [28]. After the sample was hydrolyzed in 5 mol/L hydrochloric acid solution for 24 h, the kinds and contents of amino acids were determined by automatic amino acid analyzer. Tryptophan was determined after alkaline hydrolysis. Atomic absorption spectrophotometer was selected to measure inorganic elements (K, Ca, Mg, Fe, Zn, and Mn) [29].

### 2.6. Fractionation of Crude Fucoidan from Sargassum zhangii

Crude fucoidan from *Sargassum zhangii* was purified by DEAE cellulose. The DEAE cellulose column (60 cm × 2.6 cm) was fixed vertically, and 20 mg/mL crude fucoidan solution was added. Different concentrations of NaCl solution (0, 0.5, 1, 1.5, and 2 mol/L) were used for elution. The absorbance value of fucoidan solution was detected by phenol-sulfuric acid method [30], the elution curve was drawn according to the number of tubes and absorbance value, and the same peak sample was collected according to the elution curve. The purified fucoidan was obtained by concentration, dialysis, and vacuum freeze-drying.

### 2.7. Monosaccharide Composition Analysis

According to the method of Cui et al. [31], we proceeded with a slight modification. The monosaccharide composition of purified fucoidan from *Sargassum zhangii* was determined by GC-MS with nitrile acetate derivatives. The 5 mg purified fucoidan and 4 mL 2 mol/L Trifluoroacetic acid were hydrolyzed at 100 °C for 5 h, rotated, and evaporated at 40 °C; then, 4 mL methanol was repeatedly added and evaporated 4 times. Following this, 0.5 mL pyridine and 10 mg hydroxylamine hydrochloride were added to the sample obtained in the previous step and bathed in water at 90 °C for 30 min. After cooling to room temperature, we added 0.5 mL acetic anhydride and continued to bathe at 90 °C for 30 min. At the end of the reaction, N_2_ was used to dry the solution, and 2 mL methanol was added 3 times. After extraction with chloroform and filtration with 0.22 μm filter membrane, the samples were detected by GC-MS (AOC-5000, Shimadzu, Kyoto, Japan) with Rtx-5ms column. In addition, the 5 mg each monosaccharide standard was derivatized simultaneously with the hydrolyzed monosaccharide sample.

### 2.8. Determination of Molecular Weight (Mw)

According to Wang et al. [32] and our previous method [19], size-exclusion chromatography was used to detect Mw. In short, fucoidan (ZF1, ZF2, and ZF3) and 0.5 mol/L NaCl were mixed into 10 mg/mL solution and filtered through 0.22 μm membrane and Sephacryl Smur300 HR gel column. The injection volume was 1 mL, and the flow rate was 0.6 mL/min. The Mw of standard glucan were 1.0 × 10^4^, 4.0 × 10^4^, 7.0 × 10^4^, 1.0 × 10^5^, and 1.1 × 10^5^ Da, respectively. The concentration of polysaccharide was determined by phenol-sulfuric acid [30].

### 2.9. Fourier Transformed-Infrared (FTIR) Spectrum Analysis

We ground KBr in an agate mortar, passed through a 100-mesh sieve, and dried under infrared light for 4 h. The KBr was pressed into a translucent sheet to make a blank. ZF1, ZF2, and ZF3 were weighed and pressed into tablets with KBr, respectively. ZF1, ZF2, and ZF3 were scanned by FTIR (Magna 760, Thermo Nicolet Corporation, Waltham, MA, USA) in the range of 4000–400^−1^ cm.

### 2.10. Cell Culture and Cell Viability

HepG2 cells were cultured in DMEM medium supplemented with 10%FBS and 1% penicillin-streptomycin in 5% CO_2_ constant temperature and humidity incubator. Fucoidan used in cell experiment was dissolved in culture medium DMEM. According to our previous method [33], MTT assay was used to detect cell viability. The cells with exponential growth were inoculated into the experiment. HepG2 cells were inoculated in 96-well culture plate with 1 × 10^5^ cells/mL for 24 h. Then, the cells were treated with different concentrations (0, 50, 100, 200, 400, and 800 μg/mL) of fucoidan (ZF1, ZF2, and ZF3) for 24 and 48 h. Simvastatin (Sim), a cholesterol-lowering drug, served as a positive control. After adding 5 mg/mL MTT for 4 h, the supernatant was discarded, and 150 μL DMSO was added to avoid light for 10 min. Finally, the absorbance value was detected by a microplate reader (Varioskan Flash, Thermo Scientific, Waltham, MA, USA) in 490 nm.

### 2.11. Enzyme-Linked Immunosorbent Assay (ELISA)

According to the manufacturer’s instructions, intracellular total cholesterol (TC) content was measured by a microplate reader. The cells treated with fucoidan or Sim were transferred into 1.5 mL centrifuge tube and centrifuged for 5 min at 800 rpm/min. The supernatant was discarded, and 100 μL cell lysate was added. After 2000 rpm/min centrifugation for 5 min, the supernatant was used to determine the concentration of protein and TC. The protein concentration was detected by BCA kit. According to the instructions of the TC kit, the working liquid was prepared. The standard sample of 5 mmol/L cholesterol was diluted with absolute ethanol to 1250, 625, 312.5, 156, 78, and 39 μmol/L, and 10 μL was added to 96-well plate. Then, 10 μL samples were added to the 96-well plate and 90 μL working solution was added to each well and then reacted at 37 °C for 20 min. The OD value was determined at 550 nm. The content of TC was calculated from the standard curve. The TC content was corrected by the concentration of cellular protein.

### 2.12. Statistical Analysis

All the experiments were carried out in triplicate, and the measured data were expressed as average and standard deviation. The statistical differences between the results were analyzed by one-way analysis of variance (ANOVA) and multiple comparisons using Tukey test. *p* < 0.05 was considered to be significant and was expressed by *. *p* < 0.01 was considered to be extremely significant and was expressed by **. The Origin 2021 software (OriginLab, Northampton, MA, USA) was used for drawing.

## 3. Results

### 3.1. Chemical Composition Analysis of Six Crude Fucoidans

The chemical composition of crude fucoidan from six Sargassum species IS shown in Table 1. The crude fucoidans from *Sargassum henslowianum*, *Sargassum zhangii*, *Sargassum hemiphyllum*, *Sargassum naozhouense*, *Sargassum integerrimum*, and *Sargassum wightii* were identified as HenF, ZF, HemF, NF, IF, and WF. The total sugar content of six fucoidans ranged from 22.56% to 33.37%. The highest total sugar content was ZF, and the lowest was WF. The content of fucose in HenF was the highest, as high as 9.14%. The fucose content of WF was also the lowest. The sulfate content of ZF was 13.63%, which was higher than crude fucoidan from the other five Sargassum.

### 3.2. The Ability of Six Crude Fucoidans to Bind Bile Acids

**Figure 1:**
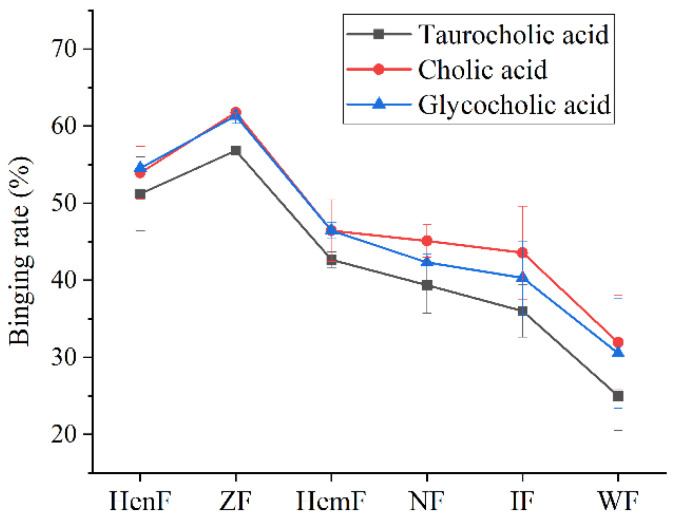
Bile acid binding rate of six *Sargassum* crude fucoidans. *Sargassum* crude fucoidans includes HenF, ZF, HemF, NF, IF, and WF, respectively, from *Sargassum henslowianum*, *Sargassum zhangii*, *Sargassum hemiphyllum*, *Sargassum naozhouense*, *Sargassum integerrimum*, and *Sargassum wightii*. Bile acid includes taurocholic acid and cholic acid and glycocholic acid.

As shown in Figure 1, the binding ability of six crude fucoidans to three cholates from high to low was ZF, HenF, HemF, NF, IF, and WF. The binding rate of ZF to taurocholic acid, cholic acid and glycocholic acid was the highest, reaching 56.82%, 61.75%, and 61.34%, respectively. The binding rate of HenF to taurocholic acid, cholic acid, and glycocholic acid was only 5.60%, 7.82%, and 6.79% lower than ZF, respectively. The ability of HemF, NF, and IF to bind bile acid was similar, and the binding rate was between 46.43% and 36.00%. The binding rate of WF to bile acids was the lowest, and the binding rate of WF to taurocholic acid, cholic acid, and glycocholic acid was 31.83%, 29.82%, and 30.82% lower than ZF, respectively. In addition, the binding rate of six crude fucoidans to glycocholic acid was lower than taurocholic acid and cholic acid.

### 3.3. Nutritional and Chemical Composition of Sargassum zhangii

Proteins, carbohydrates, ash, and dietary fiber were rich in *Sargassum zhangii* (Table 2). The contents of proteins and carbohydrates, ash, and dietary fiber were 13.97%, 70.25%, 14.54%, and 13.09% on the dry basis, respectively. The content of lipid was low, only 1.24%.

The amino acid composition and content of *Sargassum zhangii* are shown in Table 3. The protein of *Sargassum zhangii* was composed of 18 kinds of amino acids. The content of amino acid was 0.15–1.66 g/100 g protein. The content of glutamate was the highest (1.66 g/100 g protein), followed by aspartic acid (1.30 g/100 g protein). The content of cystine was the lowest, which was 0.15 g/100 g protein. The contents of amino acids and essential amino acids in *Sargassum zhangii* were 12.15 g/100 g protein and 5.40 g/100 g protein, respectively. The EAA/TAA ratio and EAA/NEAA ratio were 0.44 and 0.80, respectively.

The mineral content of *Sargassum zhangii* is shown in Table 4. *Sargassum zhangii* was rich in Ca, with a content of 1980 mg/100 g. The contents of Fe, Zn, and Mn were 85, 22, and 16.4 mg/100 g, respectively.

### 3.4. Fractionation of ZF

**Figure 2:**
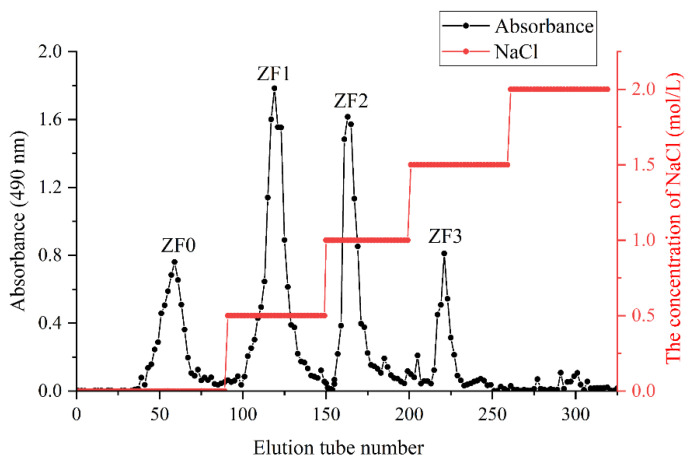
Stepwise elution curve of ZF on the DEAE cellulose 52 ion column chromatography column. Red indicates the concentration of NaCl. The fucoidans obtained from 0, 0.5, 1, and 1.5 mol/L NaCl eluents were called ZF0, ZF1, ZF2, and ZF3, respectively.

Five fractions were obtained by fractionation of crude fucoidan (ZF) by DEAE cellulose (Figure 2). They are ZF0 (0 mol/L NaCl), ZF1 (0.5 mol/L NaCl), ZF2 (1.0 mol/L NaCl), ZF3 (1.5 mol/L NaCl), and ZF4 (2.0 mol/L NaCl). Our results showed that no polysaccharides were detected when the elution phase concentration was 2 mol/L NaCl. Somasundaram et al. also found that fucoidan from *Sargassum cinereum* was fractionated by DEAE cellulose, and no polysaccharide was detected with the concentration of eluent phase of 2.0 mol/L NaCl [34]. The polysaccharides eluted from 0 mol/L NaCl were neutral polysaccharides, which were not exchanged by DEAE cellulose anion exchanger, and had less sulfate content. Therefore, ZF1, ZF2, and ZF3 were selected to analyze physicochemical characterization.

### 3.5. Chemical Composition and Molecular Weight (Mw) of ZF1, ZF2, and ZF3

As shown in Table 5, the total sugar content of ZF1, ZF2, and ZF3 was 64.77%, 68.75%, and 73.48%, respectively. The fucose content of ZF3 was the highest, as high as 26.01%. The fucose content of ZF3 was 21.07% and 8.2% higher than ZF1 and ZF2, respectively. ZF2 had the highest sulfate content, reaching 19.39%. The sulfate of ZF2 was 16.1% and 0.5% higher than ZF1 and ZF2, respectively. The Mw of ZF1, ZF2, and ZF3 were 4.026 × 10^5^, 2.893 × 10^5^ and 3.368 × 10^5^ Da, respectively. The Mw of ZF2 was the smallest, and ZF1 was the largest.

### 3.6. Analysis of Monosaccharide Composition of ZF1, ZF2, and ZF3

**Figure 3:**
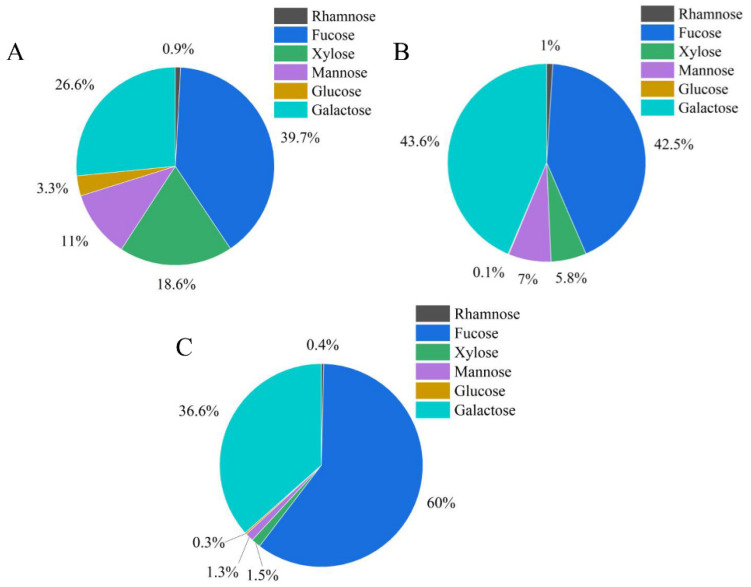
Monosaccharide composition of ZF1, ZF2, and ZF3. (**A**) Monosaccharide composition of ZF1; (**B**) monosaccharide composition of ZF2; and (**C**) monosaccharide composition of ZF3.

ZF1, ZF2, and ZF3 contained the same kinds of monosaccharides, but the contents of monosaccharides were different. ZF1, ZF2, and ZF3 all had six kinds of monosaccharides, which were rhamnose, fucose, xylose, mannose, glucose, and galactose. As shown in the Figure 3, ZF1 mainly contained fucose, galactose, xylose, and mannose, with a mass fraction of 39.7%, 26.6%, 18.6%, and 11.0%, respectively. ZF2 and ZF3 mainly contained fucose and galactose, and the content of these two monosaccharides was much higher than other monosaccharides. The content of fucose in ZF3 was the highest, reaching 60.0%. ZF2 had the highest galactose content, up to 43.6%. The fucose content of ZF3 was 17.5% higher than ZF2, while the galactose content of ZF2 was 7.0% higher than fucose.

### 3.7. FTIR Spectrum of ZF1, ZF2, and ZF3

**Figure 4:**
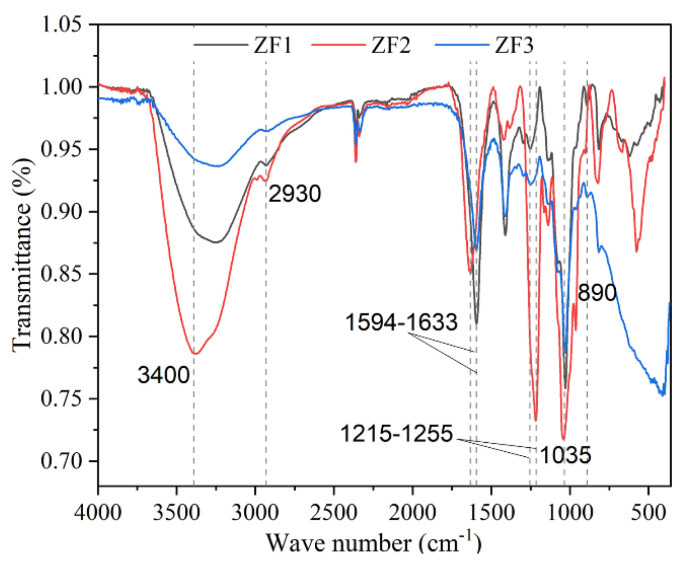
FTIR spectrum of ZF1, ZF2, and ZF3. The scanning range of FTIR spectrum is 4000–400 cm^−1^.

The FTIR spectrum of the three fractions showed the typical absorption bands of fucoidan (Figure 4). The three fucoidan FTIR spectra were basically the same, all of which had the characteristic absorption band of fucoidan. The band near 3400 cm^−1^ was caused by the O-H stretching vibration of intramolecular or intermolecular hydrogen bonds of polysaccharides. The band near 2930 cm^−1^ were assigned as C–H stretching vibrations [23]. The band at 1633 cm^−1^ was attributed to the acyl group [35]. The peak of 1594 cm^−1^ was due to the bending vibration of O–H [36]. The band at 1215–1255 cm^−1^ was attributed to the existence of sulfate group, which was a characteristic component of fucoidan. The strong absorption at 1040–1030 cm^−1^ corresponds to the C–O–C and C–O–H stretching frequency. The band of 890 cm^−1^ may indicate the existence of β-glycosidic bond between monosaccharide units.

### 3.8. The Ability of ZF1, ZF2, and ZF3 to Bind Bile Acids

**Figure 5:**
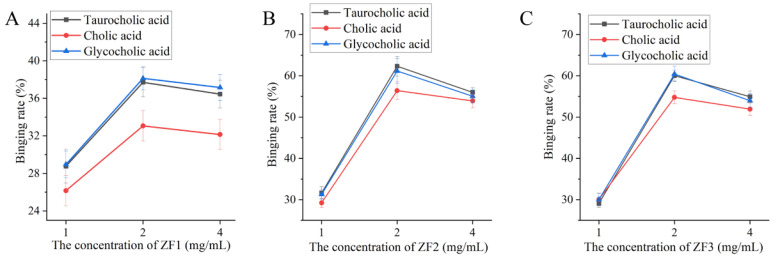
Bile acid binding rate of ZF1, ZF2,, and ZF3. (**A**) The binding rates of three different concentrations (1, 2, 4 μg/mL) of ZF1 to taurocholic acid, cholic acid and glycocholic acid; (**B**) the binding rates of three different concentrations (1, 2, and 4 μg/mL) of ZF2 to taurocholic acid, cholic acid, and glycocholic acid; (**C**) the binding rates of three different concentrations (1, 2, 4 μg/mL) of ZF3 to taurocholic acid, cholic acid, and glycocholic acid.

The result of the ability to bind to bile acids of fucoidan is shown in Figure 5. The binding ability of 2 mg/mL fucoidan to three kinds of bile acid was better than the other two concentrations (1 and 4 mg/mL). The binding ability of fucoidan to cholic acid was lower than taurocholic acid and glycocholic acid. The binding ability of ZF1 to bile acid was lower than ZF2 and ZF3. The binding rate of 2 mg/mL ZF1 to taurocholic acid, cholic acid, and glycocholic acid was 37.71%, 33.07%, and 38.13%, respectively (Figure 5A). It was similar to the ability of ZF2 and ZF3 to bind bile. The binding rate of 2 mg/mL ZF2 to taurocholic acid, cholic acid, and glycocholic acid was 62.33%, 56.41%, and 61.18%, respectively (Figure 5B). The binding rate of 2 mg/mL ZF3 to taurocholic acid, cholic acid, and glycocholic acid was 60.03%, 54.79%, and 60.45%, respectively (Figure 5C).

### 3.9. Cell Viability

**Figure 6:**
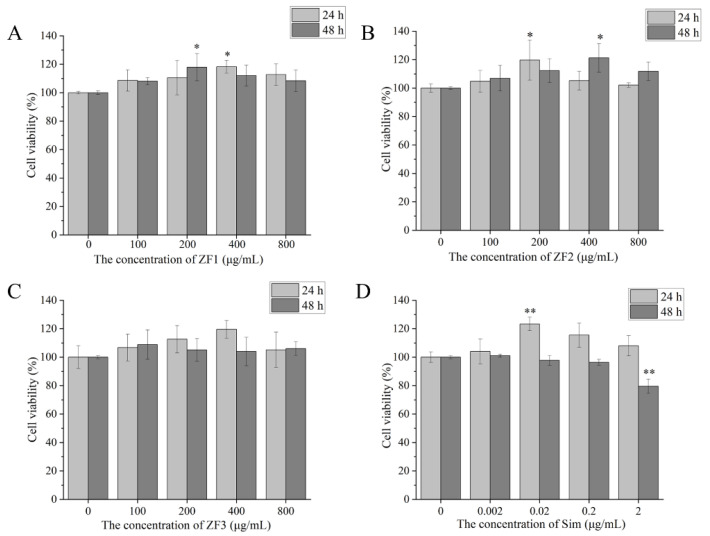
The viability of HepG2 cells treated with different concentrations of ZF1, ZF2, ZF3, and Sim for 24 h and 48 h. (**A**) The viability of HepG2 cells treated with different concentrations (0, 100, 200, 400, and 800 μg/mL) of ZF1 for 24 h and 48 h; (**B**) the viability of HepG2 cells treated with different concentrations (0, 100, 200, 400, and 800 μg/mL) of ZF2 for 24 h and 48 h; (**C**) the viability of HepG2 cells treated with different concentrations (0, 100, 200, 400, and 800 μg/mL) of ZF3 for 24 h and 48 h; (**D**) the viability of HepG2 cells treated with different concentrations (0, 0.002, 0.02, 0.2, and 2 μg/mL) of Sim for 24 h and 48 h. * indicates *p* < 0.05, and ** indicates *p* < 0.01.

As shown in Figure 6, 0–800 μg/mL ZF1, ZF2, and ZF3 had no toxicity to HepG2 cells at 24 h and 48 h. Likewise, 200 and 400 μg/mL ZF1, ZF2, and ZF3 increased cell viability. Low concentration (200 μg/mL) and high concentration (800 μg/mL) of ZF1, ZF2, and ZF3 were selected for cholesterol determination. Additionally, 0.002, 0.02, and 0.2 μg/mL Sim showed no toxicity to HepG2 cells, and2 μg/mL Sim significantly promoted cell viability at 24 h (*p* < 0.01). However, 2 μg/mL Sim decreased cell viability at 48 h. Therefore, we chose 0.2 μg/mL Sim as the positive control.

### 3.10. Intracellular TC Content

**Figure 7:**
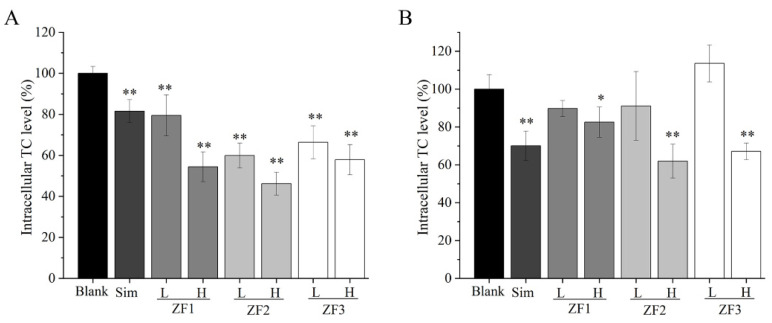
The effects of low and high concentrations of ZF1, ZF2, and ZF3 on intracellular TC content at 24 h and 48 h. (**A**) The effects of low and high concentrations of ZF1, ZF2, and ZF3 on intracellular TC content at 24 h; (**B**) the effects of low and high concentrations of ZF1, ZF2, and ZF3 on intracellular TC content at 48 h. The cells in blank group were cultured in DMEM medium without fucoidan. The L refers to a low concentration (200 μg/mL) of fucoidan, and H means a high concentration (800 μg/mL) of fucoidan. Compared to blank, * indicates *p* < 0.05, and ** indicates *p* < 0.01.

As shown in Figure 7, low and high concentrations of ZF1, ZF2, and ZF3 could reduce the content of TC in HepG2 cells at 24 h. The ability of high concentration fucoidans to reduce TC was better than low-concentration fucoidans at 24 h. ZF2 showed a better ability to reduce TC than ZF1 and ZF3. Compared with Sim, three fucoidans showed superior activity in reducing TC (Figure 7A). The ability of reducing TC of low-concentration fucoidan was worse than that of Sim at 48 h. High concentrations of fucoidan, especially ZF2, could also significantly reduce the content of TC at 48 h.

## 4. Discussion

The chemical composition of fucoidan derived from brown algae varies greatly with different species of brown algae. Ultrasonic-assisted hot water extraction was used to extract fucoidan from six *Sargassum* spp. (*Sargassum henslowianum*, *Sargassum zhangii*, *Sargassum hemiphyllum*, *Sargassum naozhouense*, *Sargassum integerrimum*, and *Sargassum wightii*) growing in Zhanjiang sea area. The chemical compositions of the six crude fucoidans were different, which was related to their different sources. The contents of total sugar and sulfate in ZF were the highest. The fucouse content of HenF was the highest. Bile acid binding experiment proved that ZF and HenF had good binding ability, which may be related to their high contents of total sugar, fucoidan, and sulfate. The sulfate content of fucoidan is the key factor to determine the efficacy of various biological activities [37]. It was reported that the antithrombotic and anticoagulant activity of persulfate fucoidan increases with the increase of sulfate content [38]. Wang et al. found that the antioxidant capacity of fucoidan increased with the increase of sulfate content [39]. There are abundant species of *Sargassum* growing near the Zhanjiang sea area, and the *Sargassum* spp. with good activity has been selected for follow-up activity study, which is helpful to improve the local utilization of the sargassum resources and bring economic effects. Therefore, *Sargassum zhangii* was selected to analyze nutritional and chemical composition. Fucoidans from *Sargassum zhangii* were purified, and the physical and chemical properties of the purified fractions and the ability to bind bile acids were studied.

*Sargassum* spp. has folk applications in human nutrition and was considered to be a rich source of vitamins, carotenoids, proteins, and minerals [6]. *Sargassum zhangii* is a kind of edible brown algae, which is endemic to the subtropical coast of China. It is mainly distributed in the sea area near Zhanjiang, especially in Leizhou Peninsula [40]. It was the first analysis of the nutritional composition and chemical composition of *Sargassum zhangii*. Our results confirmed that *Sargassum zhangii* was mainly composed of carbohydrates, protein, and ash. It was rich in insoluble dietary fiber and contained calcium, iron, zinc, and other mineral elements. The carbohydrate content of *Sargassum zhangii* was 22.52% higher than that of *Sargassum naozhouense* of the same genus [41] and 36.75% higher than *Sargassum polycystum* [42]. It was 14.68% higher than *Codium cylindricum Holmes* of different genera [43]. The protein content was similar to that of some species of the same genus, such as *Sargassum naozhouense* (11.20%) [41] and *Sargassum vulgare* (13.61%) [44]. Dietary fiber is one of the indispensable nutrients in human body [45]. It was beneficial to gastrointestinal peristalsis, lower cholesterol, and hypoglycemia and other functions [42,46]. The dietary fiber of *Sargassum zhangii* was 8.26% higher than *Sargassum naozhouense* [41]. Lipid was only 1.24%, which was as low as other *Sargassum* genera [41]. The ideal protein standard of FAO/WHO points out that in high-quality protein, the ratio of EAA/TAA should be between 0.4 and 0.6, and the ratio of EAA/NEAA should be above 0.6. The EAA/TAA ratio and EAA/NEAA ratio of *Sargassum zhangii* were 0.44 and 0.80, respectively. It belonged to high-quality protein. The Ca content of four species of seaweed (*Porphyra tenera*, *Saccharina japonicus*, *Undaria pinnatifida*, and *Sargassum fusiforme*) in Korea was 18.00–131.29 mg/100 g [47], which was lower than *Sargassum zhangii*. Moreover, the contents of Mg, Fe, and Zn were higher than four seaweeds. Therefore, it was a kind of good raw material that was high-carbohydrate, high-protein, low-fat, and rich in minerals, which had high edible value and health function.

Chromatography is an analytical technique that is widely used to separate mixtures of chemical substances into separate components [48]. In the study, DEAE cellulose was used to fractionate and purify ZF to obtain fractions ZF1, ZF2, and ZF3. Je et al. found that commercial fucoidan had a strong absorption band at 1220–1270 cm^−1^, which confirmed the large amount of sulfate group in commercial fucoidan. The FTIR spectrum of commercial fucoidan showed an absorption peak near 1035 and 1616 cm^−1^ [49]. Fauziee et al. showed that commercial fucoidan also had a wide absorption band near 3400 cm^−1^, which corresponded to O–H stretching vibrations of polysaccharides. The band range between 1220 and 1230 cm^−1^ indicated the existence of S=O stretching [50]. This is consistent with the spectral results of fucoidan we extracted. Therefore, ZF1, ZF2, and ZF3 had characteristic absorption bands of fucoidan and sulfate groups (Figure 4). However, ZF1, ZF2, and ZF3 showed different bile acid binding ability and cholesterol-lowering ability. It may be related to their chemical composition, monosaccharide composition, and Mw. Fucoidan containing high-sulfate groups showed strong activities such as antioxidant, antiviral, anticoagulant, and immunomodulatory activities [51]. The sulfate content of ZF2 (19.39%) was the highest (Table 5) and also showed better bile acid binding ability and cholesterol-lowering ability. The higher content of L-fucose is beneficial to the extensive biological activity of fucoidan [52]. The fucose content of ZF2 and ZF3 was higher than that of ZF1, and their ability to bind bile acids and lower cholesterol was also greater. Kim et al. found that low-Mw β-glucan binds more bile acids than high-Mw β-glucan [53]. The Mw of ZF2 was lower than ZF1 and ZF3, which also showed better bile acid binding ability than ZF1 and ZF3. Deng et al. showed that cholate binding capacity generally decreased with the increase of stigma maydis polysaccharides concentration [24], which was consistent with our experimental results. The binding ability of 4 mg/mL fucoidan to bile acid was lower than 2 mg/mL fucoidan (Figure 5).

Bile acid is a steroid carboxylic acid synthesized from cholesterol in the liver [54]. Cholic acid, glycocholic acid, and taurocholic acid are the main bile acids synthesized in human body [55]. Binding bile acids into polymers enhances their elimination through the fecal excretion, which builds up cholesterol metabolism in the liver to decrease plasma total cholesterol and low-density lipoprotein cholesterol levels [56]. Certain ingredients of foods and herbs bind to bile acids and prevent their absorption, resulting in some hypolipidemic effects [57]. Our results showed that fucoidan from *Sargassum zhangii* can bind to bile acid and reduce the content of TC in HepG2 cells. Hybertson et al. found that phytochemical combination PB123 treatment decreased the expression of 15 genes involved in cholesterol biosynthesis, resulting in a decrease in TC levels in HepG2 cells [58]. Huang et al. confirmed that bergamot fruit extract and neohesperidin can down-regulate the expression of 3-hydroxy-3-methylglutaryl-coenzyme A reductase, which may inhibit cholesterol accumulation by stimulating the AMPK activity of HepG2 cells [59]. We speculated that fucoidan may reduce the level of TC in HepG2 cells by changing the expression of genes and proteins involved in cholesterol synthesis. Therefore, fucoidans from *Sargassum zhangii* can be used as a promising functional supplement for lowering cholesterol and has great potential for the treatment of hyperlipidemia.

## 5. Conclusions

In our study, the potential nutritional value and cholesterol-lowering activity of *Sargassum zhangii* were studied for the first time. Our results showed that *Sargassum zhangii* contained high carbohydrates, low fats, and rich minerals. Crude fucoidan from *Sargassum zhangii* had stronger bile acid binding ability than the other five *Sargassum* species. Chemical composition, Mw, monosaccharide composition, and FTIR spectra were used to analyze the physical and chemical characterization of fractions ZF1, ZF2, and ZF3. The fractions ZF1, ZF2, and ZF3, especially ZF2, had good ability to bind bile acid and reduced the content of intracellular TC in HepG2 cells. Fucoidans from *Sargassum zhangii* have the potential to reduce cholesterol, but the potential structure–activity relationship and cholesterol-lowering mechanism need to be studied in the future.

## Figures and Tables

**Table 1:** The chemical composition of six crude fucoidans. *Sargassum* crude fucoidans includes HenF, ZF, HemF, NF, IF, and WF, respectively, from *Sargassum henslowianum*, *Sargassum zhangii*, *Sargassum hemiphyllum*, *Sargassum naozhouense*, *Sargassum integerrimum*, and *Sargassum wightii*. Chemical composition includes total sugar, fucose, and sulfate.

Crude Fucoidans	Total Sugar (%)	Fucose (%)	Sulfate (%)
HenF	31.78	9.14	12.44
ZF	33.37	7.91	13.63
HemF	28.64	8.21	10.56
NF	30.01	7.20	9.82
IF	25.42	6.40	8.59
WF	22.56	5.73	5.94

**Table 2:** Chemical composition of *Sargassum zhangii* (%, *w*/*w* on the dry basis). Chemical composition includes protein, lipid, ash, carbohydrate, and dietary fiber.

Protein	Lipid	Carbohydrate	Dietary Fiber	Ash
13.97	1.24	70.25	13.09	14.54

**Table 3:** Amino acid composition of *Sargassum zhangii* (g/100 g protein).

Amino Acid	Content	Amino Acid	Content
Methionine	0.32	Glutamate	1.66
Leucine	1.13	Glycine	0.72
Valine	0.82	Alanine	0.87
Lysine	0.67	Cystine	0.15
Isoleucine	0.68	Tyrosine	0.33
Phenylalanine	0.74	Arginine	0.59
Tryptophan	0.20	Proline	0.56
Threonine	0.60	Total amino acid (TAA)	12.15
Histidine	0.24	Essential amino acid (EAA)	5.40
Aspartic acid	1.30	EAA/TAA	0.44
Serine	0.57	EAA/NEAA	0.80

EAA, essential amino acids, including threonine, valine, methionine, isoleucine, leucine, phenylalanine, lysine, histidine, arginine, and tryptophan; NEAA, non-essential amino acids.

**Table 4:** The mineral content of *Sargassum zhangii* (g/100 g protein). The minerals tested include K, Ca, Mg, Fe, Zn, and Mn.

K	Ca	Mg	Fe	Zn	Mn
1000	1980	1080	85	22	16.4

**Table 5:** Chemical composition and Mw of ZF1, ZF2, and ZF3. Chemical composition includes total sugar, fucose, and sulfate.

Fucoidan	Total Sugar (%)	Fucose (%)	Sulfate (%)	Mw (Da)
ZF1	64.77	4.94	3.29	4.026 × 10^5^
ZF2	68.75	17.81	19.39	2.893 × 10^5^
ZF3	73.48	26.01	18.89	3.368 × 10^5^

## Data Availability

Data is contained within the article.
